# Tracing of the fecal microbiota of commercial pigs at five growth stages from birth to shipment

**DOI:** 10.1038/s41598-018-24508-7

**Published:** 2018-04-16

**Authors:** Geon Goo Han, Jun-Yeong Lee, Gwi-Deuk Jin, Jongbin Park, Yo Han Choi, Sang-Kee Kang, Byung Jo Chae, Eun Bae Kim, Yun-Jaie Choi

**Affiliations:** 1 Department of Agricultural Biotechnology and Research Institute of Agriculture and Life Science, Seoul National University, Seoul, Republic of Korea; 2 Department of Animal Life Science, Kangwon National University, Chuncheon, Gangwon-do Republic of Korea; 3 Institute of Green-Bio Science & Technology, Seoul National University, Pyeongchang, Gangwon-do Republic of Korea; 4 Division of Applied Animal Science, Kangwon National University, Chuncheon, Gangwon-do Republic of Korea

## Abstract

The intestinal microbiota affect various physiological traits of host animals such as brain development, obesity, age, and the immune system. In the swine industry, understanding the relationship between intestinal microbiota and growth stage is essential because growth stage is directly related to the feeding system of pigs, thus we studied the intestinal microbiota of 32 healthy pigs across five sows at 10, 21, 63, 93, and 147 d of ages. The intestinal microbiota were altered with growth of pigs and were separated into three distinct clusters. The relative abundance of several phyla and genera were significantly different between growth stages. We observed co-occurrence pattern of the intestinal microbiota at each growth stage. In addition, we predicted the functions of the intestinal microbiota and confirmed that several KEGG pathways were significantly different between growth stages. We also explored the relationship between the intestinal microbiota and innate factors such as the maternal effect and gender. When pigs were young, innate factors affected on construction of intestinal microbiota, however this tendency was disappeared with growth. Our findings broaden the understanding of microbial ecology, and the results will be used as a reference for investigating host-microbe interactions in the swine industry.

## Introduction

After birth, the intestinal tracts of animals are rapidly colonized by a complex of microorganisms, the intestinal microbiota. In the past, the relationship between the host and the intestinal microbiota was known as a commensalism or a parasitism; however, recent researches revealed their relationship as mutualism. With the development of high-throughput sequencing technology, culture-independent analysis of intestinal microbiota was advanced, and many researchers reported that intestinal microbiota affect the various physiological traits of hosts, such as brain development and behavior^1^, obesity^2^, hypertension^3^, age^4^, and immune functions^5,6^. In addition, it was reported that the specific bacterial species *Akkermansia muciniphila* can control diet-induced obesity in the intestine^7,8^.

Although studies of the intestinal microbiota-host interaction are actively ongoing, only a limited number of studies have been performed in farm animals such as pig, cattle, and chicken. Most of these studies are associated with the effects of treatments, such as antibiotics, prebiotics, probiotics, and feed additives, while basic studies of host physiology are scarce in farm animals. In particular, studies of the relationship between the intestinal microbiota and growth stage is essential in livestock because growth stage is directly related to the feeding system. To improve growth performances in the swine industry, feed additives such as prebiotics and probiotics have been widely used. It is important to choose the proper feed additives for the growth stage, and therefore, an understanding of the alteration of the intestinal microbiota with the growth of pigs is required. Although some researchers reported the relationship of feed and growth stage with gut microbiota in pigs^9–14^, more studies are needed to generalize the status quo.

The aim of this study was to investigate the alteration of the intestinal microbiota in various aspects such as changes in feed composition and age in pigs at different growth stages, as well as to reveal the relationship between the intestinal microbiota and innate factors such as the maternal effect and gender. To accomplish this goal, we traced the fecal microbiota of commercial pigs from birth to shipment in the same population.

## Results

### Alteration of intestinal microbiota with growth of pigs

**Figure 1:**
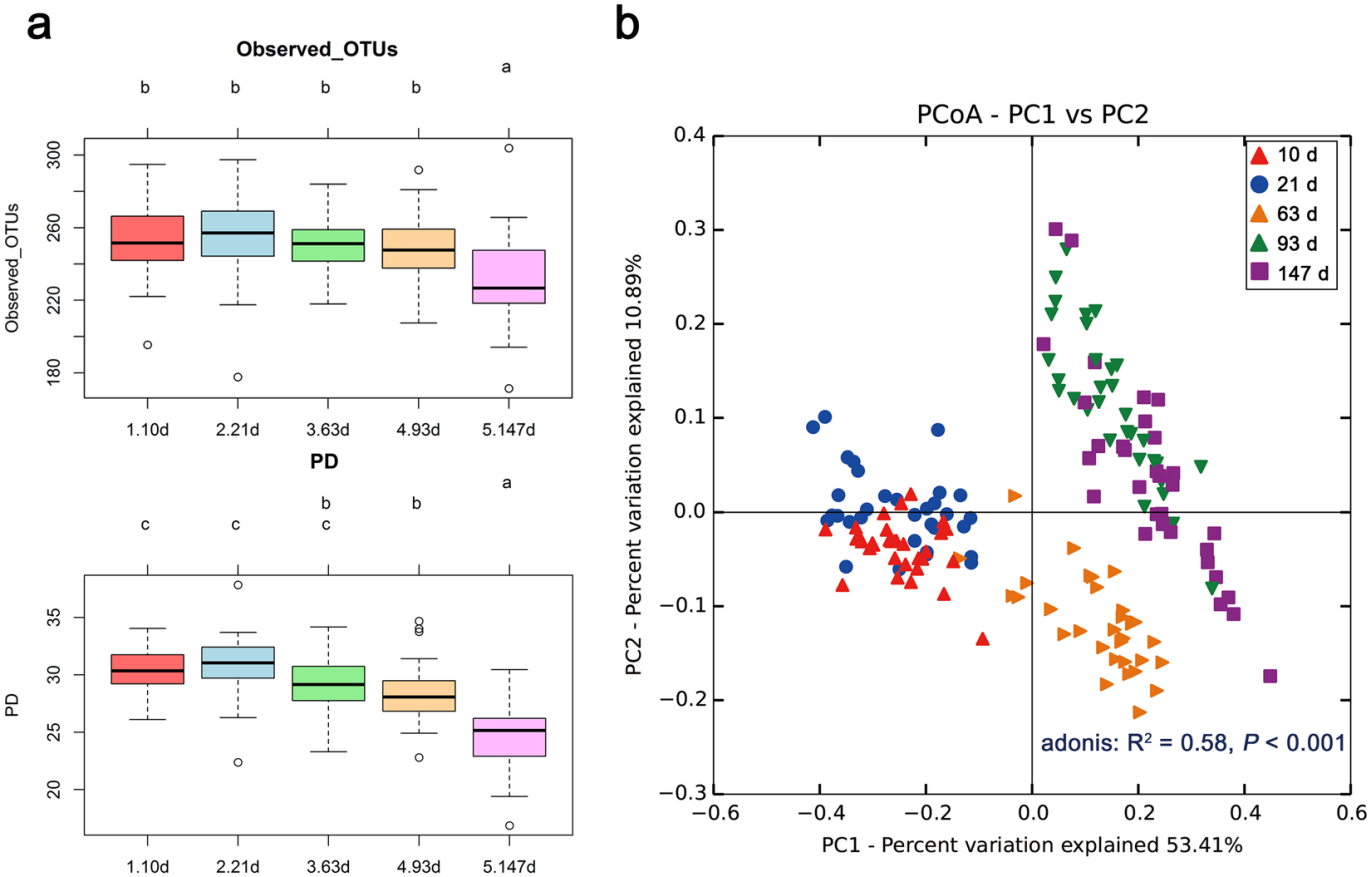
Diversity of intestinal microbiota of commercial pigs at various growth stages. (**a**) Alpha diversity indices (observed OTUs and PD) at different growth stages. One-way ANOVA with Tukey’s post hoc test was used, and different superscript letters indicate significant difference (*P* < 0.05). (**b**) Principal coordinate analysis (PCoA) plot based on weighted UniFrac distances. The effect of growth stages on microbial community was analyzed using Adonis statistical tests with 999 permutations.

We compared the microbial richness (observed OTUs) and diversity (phylogenetic diversity, PD) at various growth stages, and both the richness (*r* = −0.35, *P* < 0.001) and diversity (*r* = −0.63, *P* < 0.001) were negatively related to the age of pigs (Fig. 1a). In particular, the microbial richness and diversity were significantly lower at 147 d than other growth stages.

Principal coordinate analysis (PCoA) based on weighted UniFrac distances revealed that the intestinal microbiota of pigs were altered with growth (Fig. 1b). The Adonis test determined that the intestinal microbiota were significantly influenced by the growth of the pigs (R^2^ = 0.58, *P* < 0.001). In the PCoA plot, samples were clustered into three distinct groups: early-stage (10 and 21 d), mid-stage (63 d), and late-stage (93 and 147 d). The samples of 63 d old pigs were placed between the early and late-stage samples.

**Figure 2:**
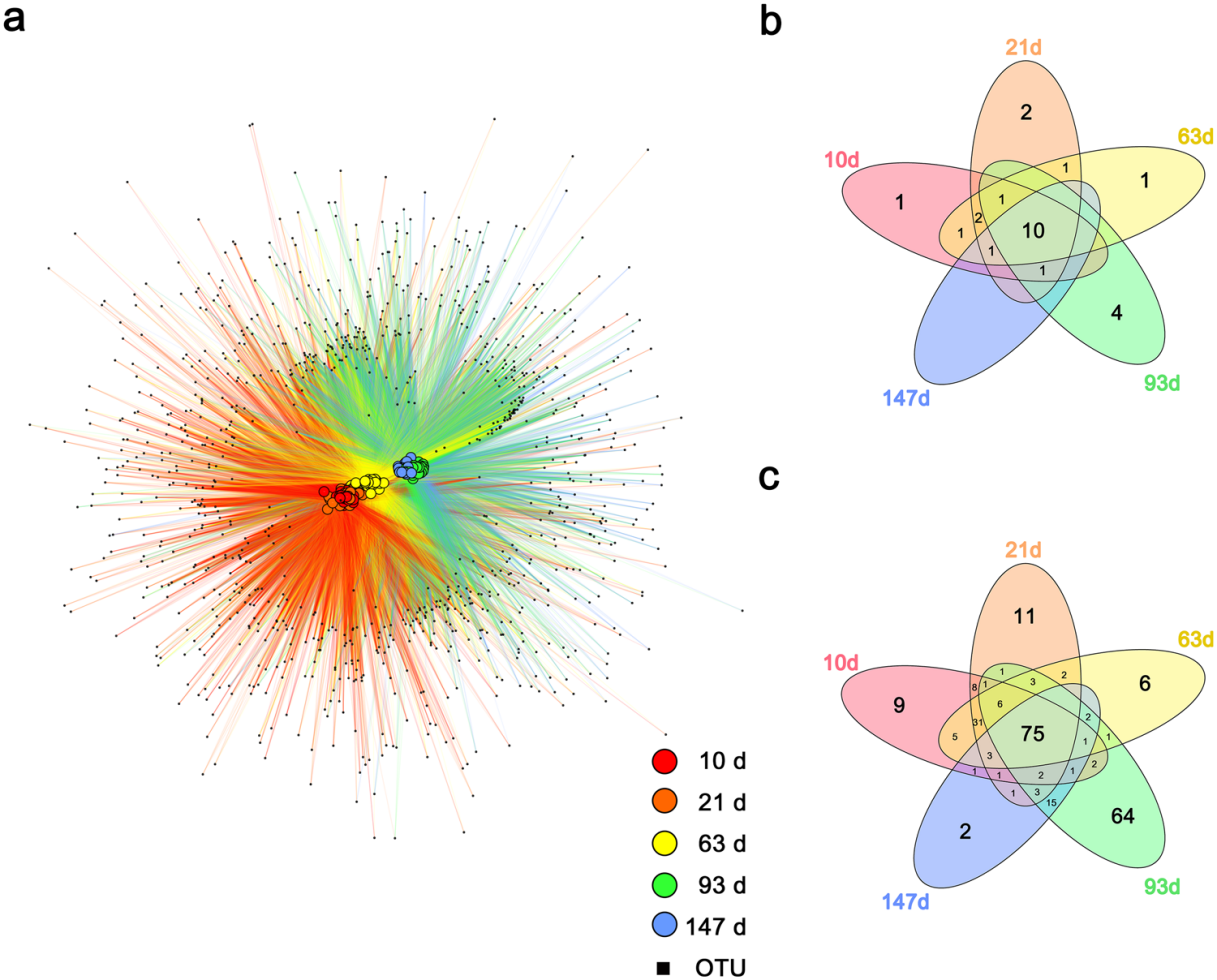
Shared taxa at different growth stages. (**a**) OTU network map of the intestinal microbiota of pigs. Edges connect sample nodes to OTU nodes detected in samples. Samples are represented as large circles with sample type designated by color, while OTUs are represented as small black rectangle. To reduce the network complexity, rare OTUs with less than 0.005% of total sequences and present in less than two samples were removed from analysis. Nodes are ordinated using an edge-weighted spring-embedded layout in Cytoscape 3.3.0. The number of phyla (**b**) and genera (**c**) shared between growth stages are shown in Venn diagrams.

Operational taxonomic unit (OTU) network analysis showed that the samples in the 10, 21, and 63 d group were more closely associated with each other than the late-stage samples (Fig. 2a). The early and mid-stage samples shared many OTUs, which was not observed in the late-stage samples. Many OTUs were shared between the late-stage samples, and these OTUs were detected only in the late-stage samples.

At the phylum level, all groups shared the following 10 phyla: *Euryarchaeota*, *Actinobacteria*, *Bacteroidetes*, *Firmicutes*, *Fusobacteria*, *Proteobacteria*, *Spirochaetes*, *TM7*, *Tenericutes*, and *Verrucomicrobia* (Fig. 2b). *Elusimicrobia* was observed only in 10 d old pigs, and *WPS-2* was observed only in 63 d old pigs. Three dominant phyla, containing more than 94% of total 16S rRNA gene sequences, were *Bacteroidetes*, *Firmicutes*, and *Proteobacteria* at 10, 21, and 63 d of age, while *Bacteroidetes*, *Firmicutes*, and *Spirochaetes* dominated at 93 d of age, and *Bacteroidetes*, *Firmicutes*, and *Euryarchaeota* at 147 d of age.

At the genus level, all groups shared 75 genera, and 31 genera were observed only in the early and mid-stage pigs, while 15 genera were observed only in the late-stage pigs (Fig. 2c). Three dominant genera, containing more than 40% of total 16S rRNA gene sequences, were *Bacteroides*, *Lactobacillus*, and *Prevotella* at 10, 21, and 63 d of age, while *Clostridium*, *Prevotella*, and an unclassified genus of family *S24-7* dominated at 93 and 147 d of age.

To determine which microbial taxa contributed to separation of the intestinal microbiota by growth stage, we performed one-way analysis of variance (ANOVA) and simple linear regression analysis, and several phyla and genera were identified (Table 1). Overall, at the phylum level, *Bacteroidetes* and *Proteobacteria* were significantly more abundant at the early-stage, while *Euryarchaeota*, *Firmicutes*, and *Spirochaetes* were significantly more abundant at the late-stage in pigs. In particular, *Proteobacteria* showed a strong negative correlation with age (*r* = −0.62, *P* < 0.001).

**Table 1:** Relative abundances of phyla and genera at various growth stages.

Taxon	Relative abundance (%)					*r* ^1^
	10 d	21 d	63 d	93 d	147 d	
**Phylum**						
*Bacteroidetes*	56.14 ± 10.16^a^	51.49 ± 10.52^ab^	50.63 ± 5.97^ac^	43.88 ± 11.09^c^	47.95 ± 11.26^bc^	−0.28**
*Euryarchaeota*	0.31 ± 0.35^a^	0.86 ± 0.76^a^	0.10 ± 0.13^a^	1.40 ± 1.07^ab^	3.10 ± 5.35^b^	0.34**
*Firmicutes*	35.48 ± 9.11^a^	38.75 ± 10.39^ab^	43.83 ± 5.60^bc^	45.04 ± 9.30^c^	44.72 ± 9.64^bc^	0.34**
*Proteobacteria*	5.14 ± 2.79^a^	4.49 ± 2.26^a^	2.77 ± 1.49^b^	2.61 ± 1.18^b^	0.93 ± 1.08^c^	−0.62**
*Spirochaetes*	0.92 ± 0.57^ab^	1.98 ± 1.37^ab^	0.55 ± 0.36^a^	5.65 ± 5.54^c^	2.57 ± 2.15^b^	0.26*
**Genus**						
*Bacteroides*	18.49 ± 8.27^a^	17.13 ± 10.70^a^	4.68 ± 3.49^b^	0.17 ± 0.42^c^	0.10 ± 0.20^c^	−0.72**
*Bifidobacterium*	0.29 ± 0.48^a^	0.35 ± 0.32^a^	0.23 ± 0.36^a^	0.00 ± 0.00^b^	0.00 ± 0.00^b^	−0.40**
*Butyricimonas*	4.92 ± 2.23^a^	2.16 ± 1.57^b^	0.59 ± 0.48^c^	0.00 ± 0.00^c^	0.00 ± 0.00^c^	−0.68**
*CF231*	0.52 ± 0.33^a^	0.36 ± 0.22^a^	1.04 ± 0.56^b^	2.30 ± 1.08^c^	1.51 ± 0.72^d^	0.55**
*Clostridium*	0.93 ± 0.68^a^	0.85 ± 0.58^a^	2.61 ± 0.69^b^	11.50 ± 4.03^c^	8.38 ± 2.21^d^	0.73**
*Desulfovibrio*	0.86 ± 0.44^a^	1.05 ± 0.50^a^	0.23 ± 0.24^b^	0.03 ± 0.05^bc^	0.00 ± 0.00^c^	−0.72**
*Dialister*	0.35 ± 0.24^a^	0.29 ± 0.29^a^	1.42 ± 0.43^b^	0.95 ± 0.46^c^	1.23 ± 0.72^bc^	0.53**
*Dorea*	0.30 ± 0.28^a^	0.26 ± 0.20^a^	0.09 ± 0.12^b^	0.03 ± 0.06^b^	0.05 ± 0.07^b^	−0.50**
*Lactobacillus*	12.66 ± 7.54^a^	12.44 ± 9.84^a^	12.25 ± 5.02^a^	2.66 ± 1.16^b^	2.77 ± 1.06^b^	−0.55**
*Megasphaera*	0.92 ± 0.44^a^	1.37 ± 0.93^a^	3.46 ± 1.60^b^	3.67 ± 1.97^b^	5.77 ± 2.94^c^	0.69**
*Parabacteroides*	2.34 ± 1.08^a^	2.66 ± 1.36^a^	0.63 ± 0.66^b^	0.82 ± 0.37^b^	0.49 ± 0.49^b^	−0.62**
*Prevotella*	13.34 ± 5.51^a^	10.96 ± 4.88^a^	32.48 ± 6.85^b^	25.83 ± 9.87^c^	34.25 ± 12.40^b^	0.65**
*Roseburia*	0.30 ± 0.21^a^	0.28 ± 0.29^a^	0.96 ± 0.33^b^	0.80 ± 0.26^b^	0.98 ± 0.30^b^	0.63**
*Ruminococcus*	1.08 ± 0.49^a^	1.33 ± 0.66^a^	0.66 ± 0.40^b^	0.60 ± 0.34^b^	0.61 ± 0.30^b^	−0.45**
*Shuttleworthia*	0.05 ± 0.11^a^	0.01 ± 0.04^a^	0.32 ± 0.30^a^	1.08 ± 0.39^b^	1.61 ± 0.96^c^	0.78**
*SMB53*	0.33 ± 0.26^a^	0.29 ± 0.21^a^	1.15 ± 0.41^b^	1.93 ± 0.59^c^	1.38 ± 0.48^b^	0.67**
*Streptococcus*	0.82 ± 0.63^a^	0.61 ± 0.56^a^	2.28 ± 0.89^b^	3.22 ± 1.00^c^	3.82 ± 1.55^c^	0.77**
f__S24-7;g__	5.64 ± 2.81	6.62 ± 4.37	4.54 ± 2.34	6.25 ± 1.47	6.68 ± 4.59	0.07

Data shown as the mean ± SD.

One-way ANOVA with Tukey’s post-hoc test was used. Within a row, different superscript letters indicate significant difference (*P* < 0.05).

^1^Pearson’s correlation coefficient was obtained from simple linear regression, and asterisk indicates significant correlation (*P < 0.01, **P < 0.001).

At the genus level, *Bacteroides*, *Bifidobacterium*, *Butyricimonas*, *Desulfovibrio*, *Dorea*, *Lactobacillus*, *Parabacteroides*, and *Ruminococcus* were significantly more abundant at the early-stage than at other stages, and they showed a strong negative correlation with age (*r* < −0.4, *P* < 0.001). *CF231*, *Clostridium*, *Dialister*, *Megasphaera*, *Prevotella*, *Roseburia*, *Shuttleworthia*, *SMB53*, and *Streptococcus* were significantly more abundant at 93 and 147 d of age than other growth stages, and they showed a strong positive correlation with age (*r* > 0.5, *P* < 0.001).

### Genera co-occurrence network at various growth stages

**Figure 3:**
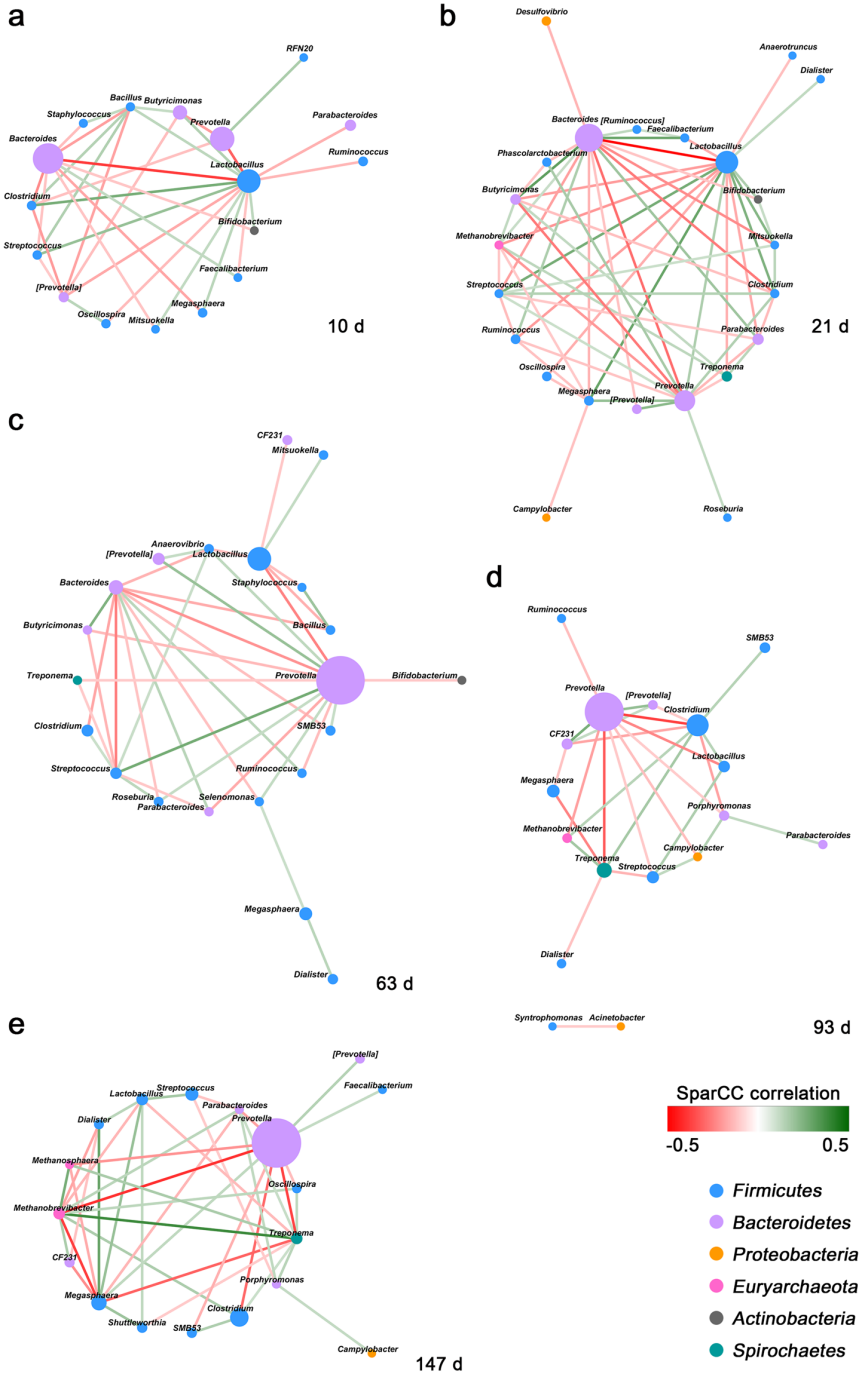
SparCC-genera co-occurrence network analysis at 10 (**a**), 21 (**b**), 63 (**c**), 93 (**d**), and 147 d of ages (**e**). The node size represents the relative abundance of genera. Node color corresponds to phylum taxonomic classification. Edges between nodes represent correlations between the nodes they connect, with edge color indicating positive (green) and negative (red) correlations, respectively, and edge shade indicating correlation magnitude.

To explore the interaction within the swine intestine microbial communities at various growth stages, we constructed a co-occurrence network at the genus level (Fig. 3).

At 10 d of age, *Lactobacillus* was positively correlated with *Clostridium* (SparCC = 0.26, *P* < 0.001), while it was negatively correlated with *Prevotella* (SparCC = −0.31, *P* < 0.001) and *Bacteroides* (SparCC = −0.38, *P* < 0.001). *Clostridium* was also negatively correlated with *Bacteroides* (SparCC = −0.22, *P* < 0.001) (Fig. 3a).

At 21 d of age, *Bacteroides* was positively correlated with *Faecalibacterium* (SparCC = 0.29, *P* < 0.001) and *Butyricimonas* (SparCC = 0.31, *P* < 0.001), while it was negatively correlated with *Lactobacillus* (SparCC = −0.49, *P* < 0.001), *Clostridium* (SparCC = −0.26, *P* < 0.001), and *Mitsuokella* (SparCC = −0.22, *P* < 0.001). *Lactobacillus* was positively correlated with *Megasphaera* (SparCC = 0.29, *P* < 0.001), *Streptococcus* (SparCC = 0.27, *P* < 0.001), *Clostridium* (SparCC = 0.25, *P* = 0.010), and *Mitsuokella* (SparCC = 0.23, *P* < 0.001) (Fig. 3b).

At 63 d of age, *Bacteroides* was positively correlated with *Butyricimonas* (SparCC = 0.25, *P* < 0.001), while it was negatively correlated with *Streptococcus* (SparCC = −0.25, *P* < 0.001), *Bacillus* (SparCC = −0.16, *P* < 0.001), and *Clostridium* (SparCC = −0.17, *P* < 0.001). *Lactobacillus* was negatively correlated with *Prevotella* (SparCC = −0.24, *P* < 0.001) (Fig. 3c).

At 93 d of age, *Prevotella* was positively correlated with *CF231* (SparCC = 0.26, *P* < 0.001), while it was negatively correlated with *Clostridium* (SparCC = −0.37, *P* < 0.001), *Treponema* (SparCC = −0.32, *P* < 0.001), and *Lactobacillus* (SparCC = −0.23, *P* < 0.001). *Lactobacillus* was positively correlated with *Clostridium* (SparCC = 0.14, *P* = 0.020) (Fig. 3d).

At 147 d of age, *Prevotella* was negatively correlated with *Clostridium* (SparCC = −0.29, *P* < 0.001), *Treponema* (SparCC = −0.36, *P* < 0.001), *Methanobrevibacter* (SparCC = −0.40, *P* < 0.001), and *Methanosphaera* (SparCC = −0.21, *P* < 0.001). *Lactobacillus* was positively correlated with *Megasphaera* (SparCC = 0.20, *P* = 0.020) and *Streptococcus* (SparCC = 0.17, *P* = 0.010). *Treponema* and *Methanobrevibacter* showed a positive correlation (SparCC = 0.38, *P* < 0.001) (Fig. 3e).

### Predicted functions of the intestinal microbiota at various growth stages

**Figure 4:**
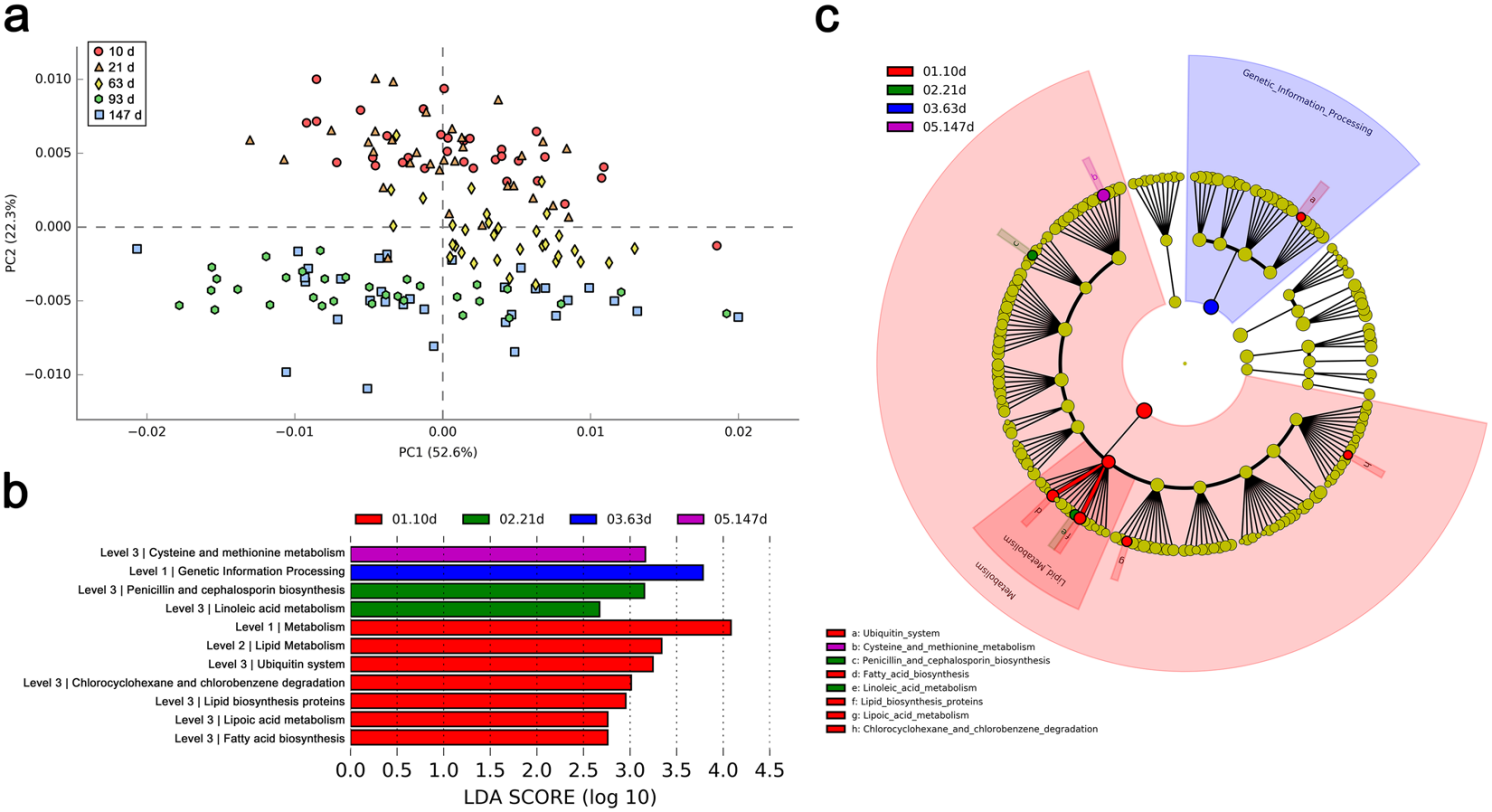
Different functions of intestinal microbiota of commercial pigs at various growth stages. Microbial functions were predicted using PICRUSt at the third level of the KEGG pathway. (**a**) Principal component analysis (PCA) plot. Histogram (**b**) and cladogram (**c**) from LEfSe analysis.

To compare the functions of the intestinal microbiota at various growth stages, the Kyoto Encyclopedia of Genes and Genome (KEGG) pathways were predicted. The prediction accuracy of PICRUSt was evaluated by the Nearest Sequenced Taxon Index (NSTI) scores, and lower scores indicate higher accuracy. The average NSTI score of 10, 21, 63, 93, and 147 d old pigs were 0.13 (±0.02), 0.13 (±0.03), 0.12 (±0.02), 0.14 (±0.01), and 0.13 (±0.02), respectively, which were in agreement with other mammal microbiota studies^15,16^. First, we performed principal component analysis (PCA) at level 3 of the KEGG pathway to observe the distribution pattern of the samples. In the PCA plot, samples were clustered into three distinct groups, and the clustering pattern was similar to the PCoA plot of the OTUs (Fig. 4a).

We next performed linear discriminant analysis (LDA) effect size (LEfSe) analysis, and several KEGG pathways were significantly different between the growth stages (Fig. 4b,c). ‘Metabolism’, ‘Lipid metabolism’, ‘Ubiquitin system’, ‘Chlorocyclohexane and chlorobenzene degradation’, ‘Lipid biosynthesis proteins’, ‘Lipoic acid metabolism’, and ‘Fatty acid biosynthesis’ pathways were predicted at significantly higher levels in the intestinal microbiota of the 10 d old pigs. ‘Penicillin and cephalosporin biosynthesis’ and ‘Linoleic acid metabolism’ pathways were predicted at significantly higher levels in the intestinal microbiota of the 21 d old pigs. ‘Genetic information processing’ and ‘Cysteine and methionine metabolism’ pathways were predicted at significantly higher levels in the intestinal microbiota of the 63 and 147 d old pigs, respectively.

### Relationship between intestinal microbiota and innate factors

To explore the relationship between the intestinal microbiota and innate factors, we investigated maternal and gender effects at each growth stage (Table 2). To analyze the maternal effect, 32 pigs were regrouped into five groups by their sows (Supplementary Table S1). Adonis test and PCoA based on weighted UniFrac distances revealed that the intestinal microbiota of pigs were separated by sow group at 10 (R^2^ = 0.28, *P* < 0.001), 21 (R^2^ = 0.43, *P* < 0.001), and 63 d (R^2^ = 0.41, *P* < 0.001). However the separation disappeared at 93 (R^2^ = 0.18, *P* = 0.125) and 147 d (R^2^ = 0.19, *P* = 0.121) (Supplementary Fig. S1).

**Table 2:** Effects of innate factors on intestinal microbiota at different growth stages.

Factor	Statistics	10 d	21 d	63 d	93 d	147 d
Maternal effect	R^2^	0.28	0.43	0.41	0.18	0.19
	*P*	<0.001	<0.001	<0.001	0.125	0.121
Gender	R^2^	0.07	0.08	0.01	0.03	0.03
	*P*	0.030	0.022	0.962	0.502	0.424

Adonis based on weighted UniFrac distances.

To analyze gender effects, 32 pigs were regrouped into two groups, male and female (Supplementary Table S1). The Adonis test and PCoA based on weighted UniFrac distances revealed that the intestinal microbiota of pigs were slightly separated by gender at 10 (R^2^ = 0.07, *P* = 0.030) and 21 d (R^2^ = 0.08, *P* = 0.022). However the separation disappeared at 63 (R^2^ = 0.01, *P* = 0.962), 93 (R^2^ = 0.03, *P* = 0.502), and 147 d (R^2^ = 0.03, *P* = 0.424) (Supplementary Fig. S2).

## Discussion

The aim of this study was to trace the alteration of the intestinal microbiota with growth in commercial pigs. To accomplish this goal, we divided the lifetime of commercial pigs into five growth stages according to feeding system, from birth to shipment. At 10 d of age, piglets were fed sow milk, and at 21 d of age, they prepared weaning, so they were fed feed and milk replacer. The 63, 93, and 147 d of age groups represented the weaned piglets, growing pigs, and finishing pigs, respectively. In addition, during the experimental period, fecal samples were obtained from identical populations at different time points. Because the environment and genetic factors can affect the microbiota^17^, this experiment was designed to reduce problems that can occur when using different populations at each age. In this regard, we used the term ‘tracing’ rather than ‘comparison’, and we evaluated the relationship between the intestinal microbiota and growth stage or feeding environment without individual variation.

In this study, we explored diversity of the intestinal microbiota at different growth stages in commercial pigs. Microbial diversity and richness of the microbial communities were reduced with the age of pigs and were significantly lower in finishing pigs (147 d old pigs) than at other growth stages. O’Toole and Jeffery reported similar results in humans^18^, but some researchers reported opposite results. Niu *et al*. reported that the abundance and diversity of the intestinal microbiota were positively correlated with age in pigs^9^, and Odamaki *et al*. reported that alpha diversity scores based on the PD whole tree, Chao1, the number of observed species, and the Shannon index increased with growth in humans^19^. The relationship between the growth and diversity of the intestinal microbiota is controversial. In fact, not only age of pigs but also complex of several factors such as feed composition, weaning, and mixing in pens, are different at each growth stage, and more studies in various environments should be performed to clarify this relationship.

To confirm alteration of the intestinal microbiota with growth, we performed PCoA based on weighted UniFrac distances. In PCoA plots (Fig. 1b), the intestinal microbial communities of commercial pigs were clustered into three distinct groups, the early (10 and 21 d), the mid (63 d), and the late-stage (93 and 147d). A similar pattern was also observed in the OTU network map (Fig. 2a). In this experiment, the differences between the three groups were age of pigs, composition of diet, and other factors. In many studies, it was reported that composition of diet is a major driving force to alter the intestinal microbiota^20–22^. The composition of the diet of commercial pigs is decided by a feeding system and is changed by the growth stage of pigs; thus, pigs within same growth stage were provided same diet (Supplementary Table S2). In addition, weaning can be a certain factor to distinguish between groups. In this experiment, pigs were weaned at 26 d of age, and weaning is one of the most stressful steps in the life of pigs^23^. During the weaning period, piglets experience various stressful events including maternal separation, change in physical environment, and transportation. They also experience rapid changes in the form of diet, from liquid milk to solid feed, and thus they must adapt to solid diet. After weaning, the structure and function of the intestine of pigs rapidly changed. Weaning of pigs induces villous atrophy, crypt hyperplasia, and the loss of digestive enzyme activity^23,24^. Gene expression levels of pro-inflammatory cytokine and heat shock proteins were altered by weaning^25,26^. In this study, the early-stage group consisted of pre-weaned piglets (10 and 21 d old pigs), and thus they were provided liquid diet or mixture with liquid and solid diet. The mid-stage group consisted of post-weaned piglets (63 d old pigs), so they had experienced weaning stress a short time ago, and thus their intestinal environment was very unstable. The late-stage group consisted of growing (93 d old pigs) and finishing pigs (147 d old pigs), which had a stable intestinal environment, because pigs adopted the dynamically changed environment, and thus, the unstable status might recover with growth. In this regard, we can assume that these factors, such as the composition of the diet and weaning, may result in intestinal microbial community alteration with the growth of pigs.

From genera co-occurrence network analysis, we revealed several relationships within the swine intestinal microbiota at various growth stages. Overall, a positive correlation was observed between the genera within the same phylum, while a negative correlation was observed between the genera belonging to the different phylum, with some exceptions. In particular, several genera of the phylum *Firmicutes* and *Bacteroidetes* showed consistent co-occurrence patterns during all growth stages. For example, *Lactobacillus* and *Clostridium*, the genera of the phylum *Firmicutes*, showed positive correlation at 10, 21, and 93 d of age. However, *Lactobacillus* was negatively correlated with *Prevotella*, the genus of the phylum *Bacteroidetes* at 10, 63, and 93 d of age, and *Clostridium* was negatively correlated with *Bacteroides* at 10, 21, and 63 d of age. It can be linked to the concept ‘Like Will to Like’ rule, wherein closely related bacteria display significant co-occurrence, although it was primarily related to ‘colonization resistance’^27^.

We confirmed that in earlier growth periods, innate factors such as the maternal effect and gender affected to intestinal microbiota of piglets although they were provided same diets. However, this tendency was disappeared with growth of the pigs. In particular, at a younger age, the impact of the maternal effect was greater than the influence of gender. We hypothesized several causes from the results. The intestinal microbiota are influenced by not only environmental factors but also host genetics^28^. In addition, piglets were raised with their sows until weaning, and thus the residential environment of piglets was similar to their sows. Therefore piglets shared not only genetic traits but also environments, including diets, with their sibling. However, after weaning, piglets were separated from their sows and raised with other piglets. In previous researches, it was suggested that the swine gut microbiota are strongly influenced by the immediate environment after separation from the sow. Thompson *et al*. reported that the gut microbiota piglets older than 31 d of ages showed significant correlation between cohabitant, but not between siblings, and this tendency was not observable in 1 or 2 week old piglets^29^. Le Floc’h *et al*. reported about the impact of environmental factors such as feed restriction and hygiene conditions on fecal microbiota of growing pigs^30^. Furthermore, commercial male pigs were castrated before 7 d of age, and thus gender discrimination might decrease after castration. For these reasons, at younger ages, the intestinal microbiota of pigs would be clustered by innate factors, particularly maternal effects, and the clustering would disappear with growth.

In this study, we explored the intestinal microbiota at various growth stages and confirmed that growth stage contributed to alteration of the intestinal microbiota in commercial pigs. From the results, we infer that age and growth environment, including composition of diet and weaning experience are crucial factors to develop the swine intestinal microbiota. The innate factors of pigs, such as the maternal effect and gender, affected on intestinal microbiota when they were young, but this tendency was disappeared with their growth. Our results broaden the understanding of microbial ecology, and these results will be useful data for the design of studies of host-microbe interactions, in particular in the swine industry.

## Methods

### Pigs and Sampling

A total of thirty-two crossbred Landrace × Yorkshire × Duroc (LYD) pigs born from five sows were raised on a local commercial farm (Gangneung, Republic of Korea) (Supplementary Table S1). The pigs were fed a commercial diet designed for each growth stage (Supplementary Table S2). The pigs were provided sow milk from birth to 9 d of age, after that they were provided a 300 g/d diet with probiotics until 25 d of age. The pigs were weaned at 26 d of age and then had access to feed and water *ad libitum*.

At 10, 21, 63, 93, and 147 d of age, fecal samples were collected from each pig and stored at −70 °C until DNA extraction was performed. All experimental procedures were performed in accordance with the Guide for the Care and Use of Laboratory Animals and approved by the Institutional Animal Care and Use Committee of Kangwon National University (KW-140509-1).

### DNA extraction and sequencing

DNA was extracted from 250 mg of each fecal sample using a NucleoSpin^®^Soil Kit (Macherey-Nagel, Düren, Germany) according to the manufacturer’s protocol and was stored at −20 °C until further analysis. The V4 region of the bacterial 16S rRNA gene was amplified from the total extracted genomic DNA using Takara Ex-taq polymerase (Takara Bio, Shiga, Japan) and universal primers (F: 5′-GGACTACHVGGGTWTCTAAT-3′ and R: 5′-GTGCCAGCMGCCGCGGTAA-3′). The amplification program consisted of 1 cycle of 94 °C for 3 min, followed by 40 cycles of 94 °C for 45 sec, 55 °C for 1 min, and 72 °C for 1.5 min, and finally, 1 cycle of 72 °C for 10 min. The amplicons were separated by agarose gel electrophoresis and purified using a QIAquick Gel Extraction Kit (Qiagen, Valencia, CA, USA).

The DNA libraries were constructed as described in our previous study^31^. The amplicons were sequenced using Illumina MiSeq. 2 × 250 bp paired-end sequencing (NICEM, SNU, Seoul, Republic of Korea). The 16S rRNA gene sequences determined in this study were deposited in the NCBI Sequence Read Archive (SRA) database with the accession number SRX2720212.

### Microbial community analysis

The microbial communities were analyzed using Quantitative Insights Into Microbial Ecology (QIIME) version 1.9.1 software^32^. The raw sequence reads were quality trimmed and demultiplexed as described in our previous study^33^. The remaining sequences were clustered into OTUs by subsampled open-reference OTU picking at 97% identity with the GreenGenes 13_8 database as the reference^34^. The OTU picking method was usearch61^35^, and the value of parameter percent_subsample was 0.1. The representative sequences were aligned using PyNAST^36^. The representative sequences were taxonomically assigned using the uclust consensus taxonomy assigner. The OTU tables were normalized to 2,700 reads per sample by single rarefaction, and rare OTUs (<0.05% relative abundance within each sample) were removed, and 4,439 OTUs were used in the downstream analysis (Supplementary Table S3).

The microbial diversity of the samples (alpha diversity) was determined using the observed OTUs and PD as richness and diversity indices, respectively. These indices were calculated from 1,770 sequence reads through rarefaction, with 10 iterations. PCoA was performed based on weighted UniFrac distances, and the effect of innate factors on the microbial community at different growth stages was evaluated using Adonis statistical tests using compare_category.py script in QIIME, with 999 permutations. The abundance of microbial taxa was expressed as a percentage of total 16S rRNA gene sequences. To analyze the effect of innate factors such as the maternal effect and gender, on intestinal microbiota at different growth stages, the OTU table was divided by growth stage using split_otu_table.py script in QIIME. One-way ANOVA and post hoc Tukey’s HSD test for multiple mean comparisons were used to find significant differences in alpha diversity and microbial taxa between the growth stages. The relationship between microbiota and the age of pigs was assessed by Pearson’s correlation coefficient (*r*) from a simple linear regression. One-way ANOVA and simple linear regression were performed using the R statistical package version 3.0.3 (R Foundation for Statistical Computing, Vienna, Austria), and significance was assumed at *P* < 0.05.

The OTU network was constructed using the make_bipartite_network.py script in QIIME. To reduce the complexity of the network, only OTUs detected in least two samples were included, and rare OTUs (<0.005% relative abundance of total sequences) were excluded from the analysis. The OTU network was visualized using Cytoscape version 3.3.0^37^.

The genera co-occurrence network was constructed from correlation coefficients between abundance of genera. The correlation coefficients were calculated using SparCC^38^ and visualized using Cytoscape version 3.3.0. Only correlations with SparCC > 0.1 or <−0.1 and *P* < 0.05 were included.

### Prediction of the functions of the microbial communities

The Phylogenetic Investigation of Communities by Reconstruction of Unobserved States (PICRUSt) version 1.0.0 was used to predict the functional profile of the microbial communities based on the 16S rRNA gene sequences obtained^15^. The OTUs that did not match with the GreenGene database were removed from the OTU table. The resulting BIOM files were normalized according to known/predicted 16S rRNA gene copy numbers, and the metagenomes were predicted using precalculated KEGG orthologs. The predicted metagenomes were collapsed into a specified level in a hierarchy using the KEGG pathway metadata. Eukaryotic and unclassified functional categories were eliminated from the analysis. PCA was performed using STAMP version 2.1.3^39^. LEfSe analyses was performed with *P* < 0.05 and LDA > 2.0 using Galaxy (https://huttenhower.sph.harvard.edu/galaxy/)^40^.

## Electronic supplementary material

Supplementary information

Supplementary Table S3
