# Supplementary material for: Tracing of the fecal microbiota of commercial pigs at five growth stages from birth to shipment
Source: Sci Rep. 2018 Apr 16;8:6012. doi: 10.1038/s41598-018-24508-7 (PMC5902624; doi:10.1038/s41598-018-24508-7)
Supplement: Supplementary file 1 — Supplementary information [file 41598_2018_24508_MOESM1_ESM.pdf]

Supplementary information

Tracing of the fecal microbiota of commercial pigs at five growth stages from birth to shipment

Geon Goo Han[1], Jun-Yeong Lee[1], Gwi-Deuk Jin[2], Jongbin Park[2], Yo Han Choi[2], Sang-Kee Kang[3], Byung Jo Chae[2], Eun Bae Kim[2,4,*], and Yun-Jaie Choi[1,*]

[1] Department of Agricultural Biotechnology and Research Institute of Agriculture and Life Science, Seoul National University, Seoul, Republic of Korea

[2] Department of Animal Life Science, Kangwon National University, Chuncheon, Gangwon-do, Republic of Korea

[3] Institute of Green-Bio Science & Technology, Seoul National University, Pyeongchang, Gangwon-do, Republic of Korea

[4] Division of Applied Animal Science, Kangwon National University, Chuncheon, Gangwon-do, Republic of Korea

[*] Corresponding authors with equal contribution

Yun-Jaie Choi: E-mail: cyjcow@snu.ac.kr

Eun Bae Kim: E-mail: itanimal@kangwon.ac.kr

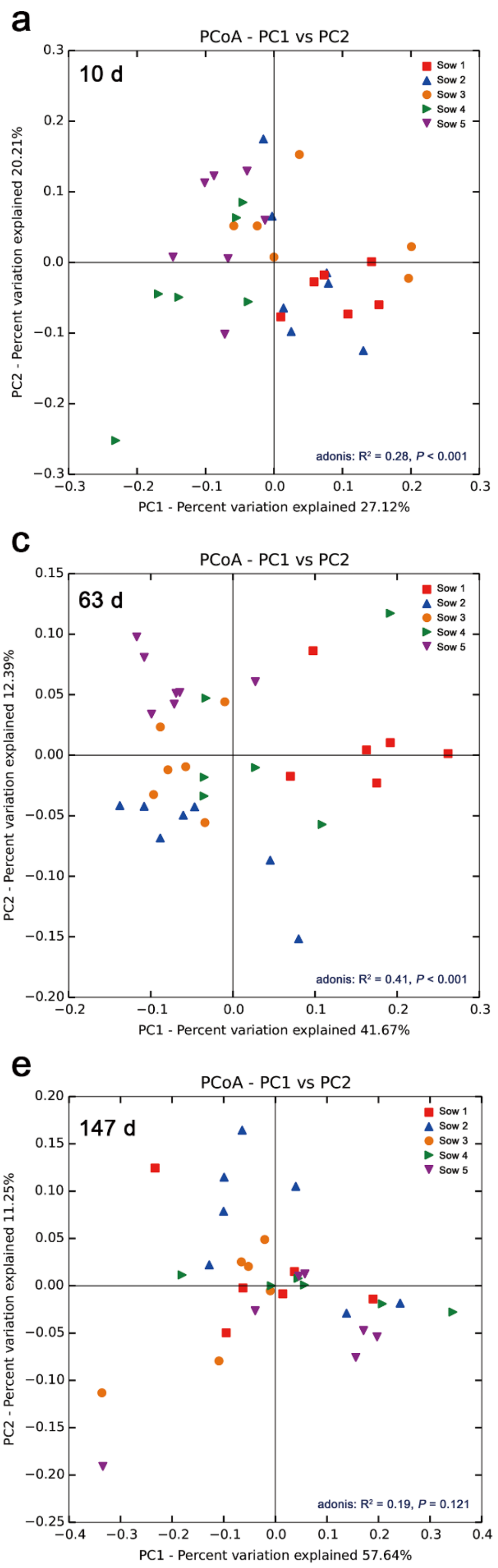

a
PCoA - PC1 vs PC2
10 d
Sow 1
Sow 2
Sow 3
Sow 4
Sow 5
adonis: R^2 = 0.28, P < 0.001
PC1 - Percent variation explained 27.12%
PC2 - Percent variation explained 20.21%
b
PCoA - PC1 vs PC2
21 d
adonis: R^2 = 0.43, P < 0.001
PC1 - Percent variation explained 37.24%
PC2 - Percent variation explained 12.22%
c
PCoA - PC1 vs PC2
63 d
adonis: R^2 = 0.41, P < 0.001
PC1 - Percent variation explained 41.67%
PC2 - Percent variation explained 12.39%
d
PCoA - PC1 vs PC2
93 d
adonis: R^2 = 0.18, P = 0.125
PC1 - Percent variation explained 45.12%
PC2 - Percent variation explained 15.33%
e
PCoA - PC1 vs PC2
147 d
adonis: R^2 = 0.19, P = 0.121
PC1 - Percent variation explained 57.64%
PC2 - Percent variation explained 11.25%

19

Figure S1. Analysis of the maternal effect on the intestinal microbiota. Principal coordinate analysis (PCoA) plot based on weighted UniFrac distances at 10 **(a)**, 21 **(b)**, 63 **(c)**, 93 **(d)**, and 147 d of ages **(e)**. The maternal effect was analyzed using Adonis statistical tests with 999 permutations.

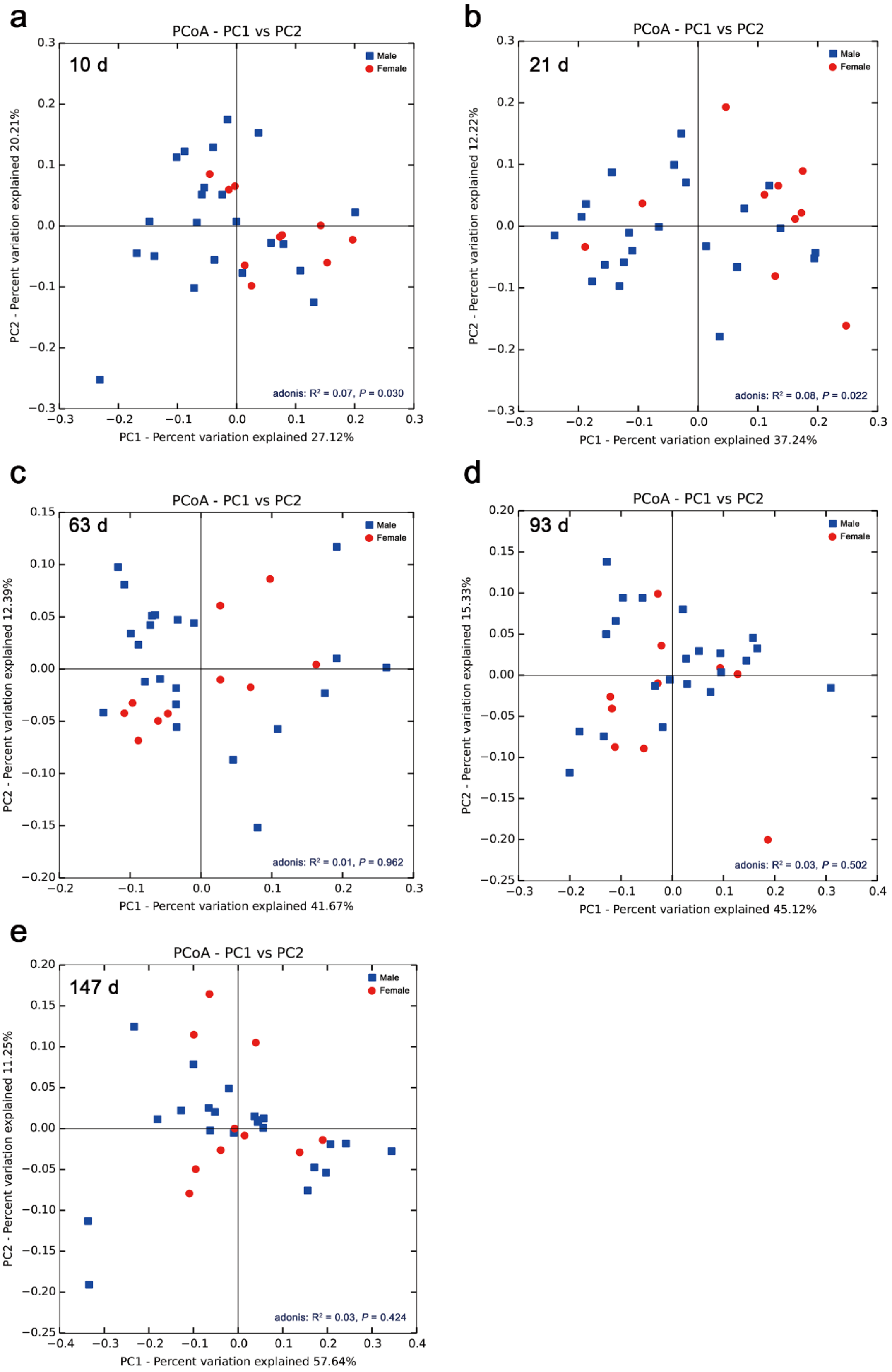
a
PCoA - PC1 vs PC2
10 d
Male
Female
adonis: R² = 0.07, P = 0.030
PC1 - Percent variation explained 27.12%
PC2 - Percent variation explained 20.21%
b
PCoA - PC1 vs PC2
21 d
Male
Female
adonis: R² = 0.08, P = 0.022
PC1 - Percent variation explained 37.24%
PC2 - Percent variation explained 12.22%
c
PCoA - PC1 vs PC2
63 d
Male
Female
adonis: R² = 0.01, P = 0.962
PC1 - Percent variation explained 41.67%
PC2 - Percent variation explained 12.39%
d
PCoA - PC1 vs PC2
93 d
Male
Female
adonis: R² = 0.03, P = 0.502
PC1 - Percent variation explained 45.12%
PC2 - Percent variation explained 15.33%
e
PCoA - PC1 vs PC2
147 d
Male
Female
adonis: R² = 0.03, P = 0.424
PC1 - Percent variation explained 57.64%
PC2 - Percent variation explained 11.25%

Figure S2. Analysis of the gender effect on intestinal microbiota. Principal coordinate analysis (PCoA) plot based on weighted UniFrac distances at 10 **(a)**, 21 **(b)**, 63 **(c)**, 93 **(d)**, and 147 d of age **(e)**. The gender effect was analyzed using Adonis statistical tests with 999 permutations.

Table S1. Sample information in this study

Individual	Sow	Gender
Pig1	Sow1	Male
Pig2	Sow1	Male
Pig3	Sow1	Female
Pig4	Sow1	Female
Pig5	Sow1	Male
Pig6	Sow1	Female
Pig7	Sow2	Female
Pig8	Sow2	Female
Pig9	Sow2	Male
Pig10	Sow2	Female
Pig11	Sow2	Male
Pig12	Sow2	Female
Pig13	Sow2	Male
Pig14	Sow3	Male
Pig15	Sow3	Male
Pig16	Sow3	Male
Pig17	Sow3	Female
Pig18	Sow3	Male
Pig19	Sow3	Male
Pig20	Sow4	Male
Pig21	Sow4	Male
Pig22	Sow4	Male
Pig23	Sow4	Female
Pig24	Sow4	Male
Pig25	Sow4	Male
Pig26	Sow5	Male
Pig27	Sow5	Female
Pig28	Sow5	Male
Pig29	Sow5	Male
Pig30	Sow5	Male
Pig31	Sow5	Male
Pig32	Sow5	Male

Table S2. Composition of the commercial diet of the pigs

Item	21 d[a]	63 d	93 d	147 d
Feed				
Metabolizable energy, kcal/kg	3,850	3,330	3,330	3,330
Crude protein, %	19.67	18.50	16.00	16.00
Crude fat, %	9.27	6.05	6.50	6.50
Crude fiber, %	0.91	3.30	3.40	3.40
Crude ash, %	6.03	5.30	5.40	5.40
Calcium, %	0.96	0.79	0.86	0.86
Phosphorous, %	0.57	0.60	0.50	0.50
Lysine, %	1.64	1.20	0.90	0.90
Methionine, %	0.64	0.40	0.30	0.30
Threonine, %	0.96	0.78	0.64	0.64
Tryptophan, %	0.23	0.24	0.17	0.17
Citric acid, %		0.20	0.20	0.20
Tylosin phosphate, %		0.01	0.01	
Sulfamethazine sodium, %		0.01	0.01	
Milk replacer				
Digestible energy, kcal/kg	4,200			
Crude protein, %	21.00			
Crude fat, %	15.00			
Crude fiber, %	3.00			
Crude ash, %	11.00			
Calcium, %	0.30			
Phosphorous, %	1.50			
Lysine, %	1.30			
Methionine, %	0.30			
Threonine, %	1.00			
Tryptophan, %	0.26			
Probiotics				
Lactobacillus fermentum, CFU/d	5×10^{7}			
Bacillus subtilis, CFU/d	5×10^{6}			
Saccharomyces cerevisiae, CFU/d	5×10^{6}			

[a]21 d old piglets were provided mixture (300 g/d) with equal weight ratio of a feed and milk replacer, and probiotics
